# Heart Failure With Reduced Ejection Fraction Polypill Implementation Strategy in India: A Convergent Parallel Mixed Methods Study

**DOI:** 10.5334/gh.1348

**Published:** 2024-08-26

**Authors:** Anubha Agarwal, Raji Devarajan, Salva Balbale, Aashima Chopra, Dorairaj Prabhakaran, Mark D. Huffman, Lisa R. Hirschhorn, Padinhare P. Mohanan

**Affiliations:** 1 Division of Cardiology, Department of Medicine and Global Health Center, Washington University in St. Louis, St. Louis, MO, USA; 2 Global Antibiotic Research and Development Partnership, New Delhi, Delhi, India; 3 Department of Medicine, Northwestern University Feinberg School of Medicine, Chicago, IL, USA; 4 Center of Innovation for Complex Chronic Healthcare, Health Services Research and Development, Edward Hines, Jr. Veterans Affairs Hospital, Hines, IL, USA; 5 Department of Preventive Medicine, Northwestern University Feinberg School of Medicine, Chicago, IL, USA; 6 Centre for Chronic Disease Control, New Delhi, Delhi, India; 7 The George Institute for Global Health, University of New South Wales, Sydney, New South Wales, Australia; 8 Department of Medical Social Sciences, Northwestern University Feinberg School of Medicine, Chicago, IL, USA; 9 WestFort Hi-Tech Hospital, Thrissur, Kerala, India

**Keywords:** heart failure with reduced ejection fraction, India, mixed methods

## Abstract

**Introduction:**

A polypill-based implementation strategy has been proposed to increase rates of guideline-directed medical therapy (GDMT) in patients with heart failure with reduced ejection fraction. This has the potential to improve mortality and morbidity in India and undertreated populations globally.

**Methods:**

We conducted a convergent parallel mixed methods study integrating quantitative data from stakeholder surveys using modified implementation science outcome measures and qualitative data from key informant in-depth interviews. Our objective was to explore physician, nurse, pharmacist, and patient perspectives on a HFrEF polypill implementation strategy in India from January 2021 to April 2021. Quantitative and qualitative data were integrated to develop an Implementation Research Logic Model.

**Results:**

Among 69 respondents to the stakeholder survey, there was moderate acceptability (mean [SD] 3.8 [1.0]), appropriateness (3.6 [1.0]), and feasibility (3.7 [1.0]) of HFrEF polypill implementation strategy. Participants in the key-informant in-depth interviews (n = 20) highlighted numerous relative advantages of the HFrEF polypill innovation including potential to simplify medication regimens and improve patient adherence. Key relative disadvantages elucidated, include concerns about side effects and interruption of multiple GDMT medications due to polypill discontinuation for side effects or hospitalizations. Based on this data, the proposed implementation strategies in the Implementation Research Logic Model include 1) HFrEF polypills, 2) HFrEF polypill initiation, titration, and maintenance protocols, and 3) HFrEF polypill laboratory monitoring protocols for safety which we postulate will lead to desired clinical and implementation outcomes through multiple mechanisms including increased medication adherence to a single pill.

**Conclusion:**

This study demonstrates that a HFrEF polypill-based implementation strategy is considered acceptable, feasible, and appropriate among healthcare providers in India. We identified contextually relevant determinants, strategies, mechanism, and outcomes outlined in an Implementation Research Logic Model to inform future research to improve heart failure care in South Asia.

## Introduction

Heart failure is a leading global public health problem and clinical outcomes of patients with heart failure with reduced ejection fraction (HFrEF) remain poor (1,2,3). Guideline-directed medical therapy (GDMT) including beta-blockers, renin-angiotensin-system inhibitors, mineralocorticoid receptor antagonists, and sodium glucose co-transporter 2 inhibitors improve mortality and morbidity of HFrEF patients (4). Observational registries in India including the Trivandrum Heart Failure Registry and the National Heart Failure Registry of India demonstrate less than half of eligible HFrEF patients receive GDMT, revealing a key target for intervention to improve clinical outcomes. Increasing GDMT rates is one of the most cost-effective interventions and a global health system priority (5). Increasing GDMT rates also aligns with the national priorities of heart failure research in India outlined by leaders in medicine and science representing the Indian Council of Medical Research, National Centre for Advanced Research and Excellence in Heart Failure, International Academy of Cardiovascular Sciences, and the Heart Failure Association of India (6).

A HFrEF polypill-based implementation strategy has been proposed to increase GDMT rates and subsequent mortality and morbidity of undertreated HFrEF patients in India and globally (7,8). There are ongoing clinical trials in the United States (NCT06029712, NCT04633005) and planned clinical trials in South Asia (SLCTR/2024/003) to evaluate the efficacy and safety of HFrEF polypills. Successful implementation of novel innovations such as HFrEF polypills requires identification of key determinants including facilitators and barriers, contextually-relevant implementation strategies, and evaluation of the process of implementation (9). Mixed methods research leveraging insights from both qualitative and quantitative data is particularly useful in this context. As part of formative research for a National Heart, Lung, and Blood Institute funded randomized trial of a HFrEF polypill in South Asia, we aim to understand diverse perspectives on heart failure care and context of a HFrEF polypill-based implementation strategy using mixed methods to create an Implementation Research Logic Model to inform future research to improve heart failure trials and care in South Asia and beyond (8).

## Methods

We conducted a convergent parallel mixed methods study integrating quantitative data from stakeholder surveys and qualitative data from key informant in-depth interviews to explore physician, nurse, pharmacist, and patient perspectives on a HFrEF polypill implementation strategy in India from January 2021 to April 2021. The study was approved by the institutional review board at Northwestern University (Chicago, United States), ethics committee at Cardiological Society of India-Kerala Chapter (Kochi, India), and ethics committee at Westfort Hi-Tech Hospital (Thrissur, Kerala). All participants provided consent prior to participation in the study, and their confidentiality was ensured throughout the research process. The study team includes members from India and the United States with diverse expertise including clinical cardiology, public health, qualitative research, and implementation science.

### Stakeholder survey data collection and analysis

We administered a structured survey electronically via email to cardiologists who are members of the Cardiological Society of India, the largest professional organization of cardiologists in India. Survey data was collected using an electronic data capture system (REDCap) to ensure data confidentiality. No incentive was provided to survey respondents to participate. The electronic survey used validated, modified implementation outcome measures (Acceptability of Intervention Measure [AIM], Intervention Appropriateness Measure [IAM], and Feasibility of Intervention Measure [FIM]) to assess acceptability, appropriateness, and feasibility of the proposed HFrEF polypill implementation strategy in India (**Supplement: Appendix 1**) (10). Acceptability is the perception that the innovation is agreeable, appropriateness is the perceived fit for a practice setting, and feasibility is defined as the extent to which a new innovation can be successfully implemented. These implementation outcome measures (AIM, IAM, FIM) have been used in other contexts to understand acceptability, appropriateness, and feasibility of a new innovation or intervention. Likert scale values range from 1 (completely disagree) to 5 (completely agree). We report means with standard deviations for each measure item.

### Key informant in-depth interviews data collection and analysis

We used a purposive sampling frame to select an initial sample of participants with diverse roles and experiences in the care of HFrEF patients or were patients themselves in Kerala, India where our team has led previous research. We then used a snowballing sampling technique to recruit additional participants with increasing variability until we achieved theoretical saturation at which no novel concepts emerged. We conducted the key informant in-depth interviews using remote tele- and video-conferencing methods such as zoom by our two team members trained in qualitative research methods (AA, RD). We developed and used a semi-structured interview guide based on the Consolidated Framework for Implementation Research (CFIR) and used probing techniques to explore specific areas in depth (**Supplement: Appendix 2**) (11,12). The qualitative data were audiotaped, transcribed verbatim, and reviewed for accuracy. Transcripts were analyzed using simultaneous deductive and inductive content analysis using an iterative directed approach guided by the CFIR domains. We developed a codebook and organized participants’ responses by the corresponding codes using Dedoose software (v8.0.42, Manhattan Beach, US). Lastly, we synthesized participants’ responses across codes to reflect themes adhering to Consolidated Criteria for Reporting Qualitative Research (COREQ) standards (**Supplement: Appendix 3**) (13).

### Mixed methods integration

Quantitative and qualitative findings were integrated to describe key themes from physician, nurse, pharmacist, and patient perspectives on a HFrEF polypill implementation strategy in India. Findings from each method were reviewed side-by-side with a focus on identifying findings that complemented, added to, or conflicted with one another using a matrix approach to develop an Implementation Research Logic Model (14).

## Results

### Stakeholder survey

**Figure 1:**
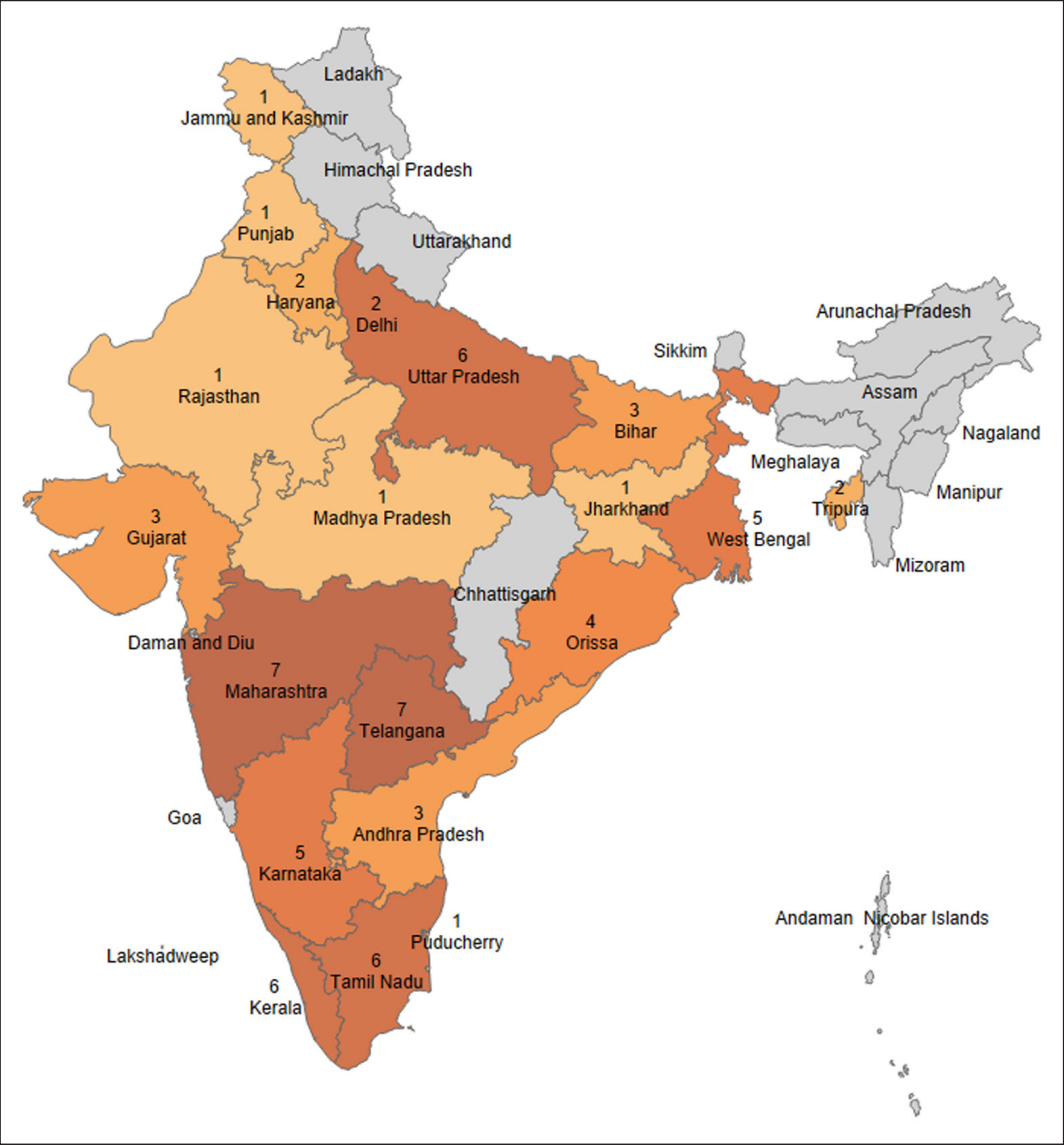
Geographic representation of stakeholder survey respondents by state or union territory in India. **Legend:** Map depicting states and union territories represented by stakeholder survey respondents (total 68 participants, 1 participant excluded due to living outside of India).

A total of 69 respondents participated in the electronic structured stakeholder survey. We excluded one respondent who reported being based outside of India, resulting in a sample of 68 respondents (Table 1). The mean (SD) age was 55.2 (11.9) years, and most were male (n = 62, 91%) cardiologists (n = 63, 93%) working in private healthcare settings (n = 51, 75%). The respondents represented various (n = 20/36, 56%) states and union territories in India (Figure 1). There was moderate acceptability (mean [SD] 3.8 [1.0]), appropriateness (3.6 [1.0]), and feasibility (3.7 [1.0]) of the proposed HFrEF polypill implementation strategy (Table 2). Most respondents noted that taking multiple pills daily is a large problem (n = 39, 57%) for their patients with HFrEF. The most important characteristics of the HFrEF polypill for patients, based on respondent perceptions, were lower cost (n = 57, 83%) and higher efficacy (n = 36, 52%, Table 2).

**Table 1:** Stakeholder survey and key informant in-depth interview participant characteristics.


PARTICIPANT CHARACTERISTICS	N (%)

**Stakeholder survey**	N = 68

Age, mean (SD), years	55.2 (11.9)

Male	62 (91.2)

Job	

Cardiologist	63 (92.6)

Internist/Family Practice	4 (5.9)

Other	1 (1.5)

Years working in healthcare, mean (SD)	28.7 (11.6)

Type of healthcare setting	

Public	16 (23.5)

Private	51 (75.0)

Other	1 (1.5)

**Key informant in-depth interviews**	N = 20

Male	15 (75)

Stakeholder type	

Cardiologist	8 (40)

Nurse	4 (20)

Pharmacist	2 (10)

Patient	6 (30)

Type of healthcare setting	

Public	6 (30)

Private	14 (70)


**Table 2:** Results of HFrEF polypill stakeholder survey with modified implementation science outcome measures.


MEASURES	SCORE, MEAN (SD)*

**Adapted Acceptability of Intervention Measure (AIM)**	

1. A HFrEF polypill meets my approval.	3.6 (1.1)

2. A HFrEF polypill would be appealing to my patients.	3.9 (1.0)

3. I like the idea of a HFrEF polypill.	3.8 (1.2)

4. I think I would be able to use a HFrEF polypill in my clinical practice.	3.8 (1.1)

**Total:**	3.8 (1.0)

**Adapted Intervention Appropriateness Measure (IAM)**	

1. A HFrEF polypill seems fitting for my patients.	3.6 (1.1)

2. A HFrEF polypill seems suitable for my patients.	3.6 (1.1)

3. A HFrEF polypill seems applicable to my patients.	3.7 (1.1)

4. A HFrEF polypill seems like a good match for my patients.	3.5 (1.1)

**Total:**	3.6 (1.0)

**Adapted Feasibility of Intervention Measure (FIM)**	

1. I welcome HFrEF polypill as an additional treatment option for my patients with HFrEF.	3.6 (1.1)

2. Using HFrEF polypills for my patients seems possible once developed.	3.7 (1.0)

3. Using a HFrEF polypill seems doable for patients.	3.7 (1.1)

4. A HFrEF polypill seems easy to use for me and my patients.	3.9 (1.0)

**Total:**	3.7 (1.0)

**Additional survey questions:**	**Responses, N = 68**

1. How much of a problem is taking multiple pills daily for your patients with HFrEF?	n (%)

Large problem	39 (57.3)

Moderate problem	10 (14.7)

Minor problem	18 (26.5)

Not a problem	1 (1.5)

2. What are the most important characteristics of a HFrEF polypill that your patients will care about?**	n (%)

Size of the HFrEF polypill	28 (40.6)

Cost of the HFrEF polypill	57 (82.6)

Side effects of the HFrEF polypill	27 (39.1)

Once daily dosing of the HFrEF polypill	32 (46.4)

Efficacy of the HFrEF polypill	36 (52.2)


*The Likert scale ranges from 1–5 and higher scores indicate greater acceptability, appropriateness, or feasibility. **Respondents had the option to select multiple answers.

### Key informant in-depth interviews

A total of 20 people participated in the key informant in-depth interviews, each lasting for 30–60 minutes (Table 1). Most were male (n = 15, 75%) and worked in private healthcare settings (n = 14, 70%). The participant group included 8 cardiologists, 4 nurses, 2 pharmacists, and 6 patients, reflecting diverse perspectives. Key themes are presented below with representative quotes.

#### Facilitators and barriers of heart failure care

Participants identified several key facilitators to heart failure care including a supportive care network (e.g., spouse, children, and other family members), nurse practitioners, and community health workers. Physicians and nurses identified compassionate care and cost as important facilitators to improving patient adherence to medications.

We have seen from our experience for the past 20 years in this hospital where we run the cardiac care unit, that once we have empathy and compassion for patients and medicines are economical to patients, then they are satisfied with their care, they comply with medications, and they improve a lot, lot, lot, lot. (Nurse)

Participants identified several key barriers to heart failure care including patient-level challenges related to medication cost and the burden of taking multiple medications daily. Cardiologists highlighted challenges such as lack of patient understanding of the disease process including the importance of adherence to medications, fragmented care without consistent outpatient follow-up, and busy outpatient clinics with limited time to initiate and titrate medications. Several cardiologists noted that their patients’ GDMT is discontinued by general medicine physicians in the community due to lack of understanding and perceived adverse effects.

Most of the cardiologists know about the importance of GDMT but at the level of physicians, especially pure general practitioners where there isn’t much of academic activity, not much of getting themselves up to date, GDMT is discontinued because for them an angiotensin converting enzyme inhibitor (ACEi) or an angiotensin receptor blocker (ARB) is basically an anti-hypertensive drug or a beta blocker is an anti-hypertensive drug. So, they can’t really grasp why this drug is being increased even though the blood pressure is only 85 or 90 systolic. (Cardiologist)

#### Treatment approach to heart failure management

Most of the cardiologists reported similar approaches to clinical management of patients with HFrEF. They preferred to start small doses of multiple GDMT drugs simultaneously depending on key clinical indices such as blood pressure, renal function, and potassium level.

Yeah, right. So, usually what I do is, if the patients’ blood pressure is reasonable, I try to start both a renin angiotensin system (RAS) blocker and a beta blocker simultaneously. Small doses of both together, I usually start, I prefer an angiotensin converting enzyme inhibitor (ACEi). I personally, rather than an angiotensin receptor blocker (ARB), I prefer an ACEi to start with. But if the BP is low and I am not very sure whether I’ll be able to start both together, then I prefer a beta blocker first for the simple reason that beta blockers are less hypo-tensive than RAS blockers. So, I start with beta blockers, once they are stabilized, I go to an ACEi. (Cardiologist)

Many cardiologists reported starting a mineralocorticoid receptor antagonist (MRA) last, either right before hospital discharge or in outpatient settings. Few patients, accessing care in the public healthcare sector, are able to afford angiotensin receptor/neprilysin inhibitors (ARNI). Sodium glucose cotransporter-2 inhibitor (SGLT2i) use was reported to be low, as the evidence of benefit of these drugs for HFrEF patients was just emerging during the time of data collection.

#### Innovation: HFrEF polypill

Numerous relative advantages of a HFrEF polypill-based approach were identified compared to the traditional sequential GDMT initiation and titration approach including potential perceived cost savings and improvement in medication adherence. Patients particularly highlighted the most important relative advantage of a HFrEF polypill to them is simplification of care with one pill daily in lieu of multiple pills for their heart failure care.

It will be good, if I have to go anywhere, we just have to take one tablet with us and we will not forget to eat it, so there will be many advantages and we can keep it in the pocket also. (Patient)

Numerous relative disadvantages of a HFrEF polypill-based approach were also identified including discontinuation of multiple components of GDMT if the HFrEF polypill were discontinued for any reason (e.g., hospitalization, side effects, self-discontinuation). Cardiologists expressed concern regarding additive adverse side effects including hyperkalemia, hypotension, and acute kidney injury with a HFrEF polypill.

Major disadvantage is this problem only, of the other side of compliance. If there is a side effect and the patient starts coughing, he will stop all the three drugs, so that is the major disadvantage. Second, again, in borderline patients where the three most important things are kidney function, potassium and blood pressure. So, if these three are borderline and if you give a combination pill, it’s quite possible that you may land up in trouble, at least in some patients you may land up in trouble. (Cardiologist)

Some cardiologists noted that the evidence-base for the individual components of GDMT is strong and supports a HFrEF polypill innovation. Others highlighted the importance of integrating HFrEF polypills into clinical care guidelines to increase innovation adoption, sustainment, and scale-up. Importantly, there are existing fixed-dose combination drugs for cardiovascular disease in India which may increase uptake of a HFrEF polypill.

Well, as I understand, all the three trade drugs that you are going to give, all already have data in heart failure. The only thing is that whether the combination behaves similarly or may be better is the only question. Theoretically, if you ask me personally, I don’t think, except for the costs, I may be not much worried about the data because we already have sufficient data with MRA, RAS blockers as well as beta blockers. (Cardiologist)

I think it should be a good study. I think you should be able to pull it off. Only problem will be the dose titration part. And then that will be, I think, the most important thing. I think what drugs you choose is important. Ramipril with Metoprolol already the combinations are available. So even I think people have now come up with Ramipril with Bisoprolol combinations in India. (Cardiologist)

Most cardiologists preferred initiating a HFrEF polypill as a substitution indication in stable outpatients who are tolerating individual doses of GDMT as opposed to de novo initiation in patients hospitalized for acute heart failure who have not previously taken any components of GDMT.

I will follow-up patients in the outpatient department and when the patient is relatively stable with comfortable doses of ACEi, beta blocker, aldosterone inhibitor and they have stable doses, then we can switch over to the polypill because then we know the usual renal function status, usual electrolyte balance and the usual dose that the patient is tolerating. For me, that’s the most convenient time to switch over to the polypill. Then we will give the supply kit to them because then the patient will need to take only one drug at one time and not need to be in contact with me and they can continue with that. (Cardiologist)

#### Outer and inner setting

The outer setting is the setting where the inner setting exists, and the inner setting is where the innovation is implemented (11). In the outer setting, patients highlighted the important role of alternative health treatments including Ayurvedic medicine as part of their socio-cultural beliefs about health and healthcare, a potential barrier to acceptability of HFrEF polypills. Nurses highlighted the importance of the doctor, specifically the cardiologist, in guiding whether patients are open to participating in clinical research. Cardiologists emphasized the potential negative influence of previous historical polypill trials for prevention of atherosclerotic cardiovascular disease on implementation of a HFrEF polypill in India.

Now there was some controversy about some polypill. I think it was against the Polycap. There were some newspaper reports or something like that and how it was tested in Kerala. When there is a drug trial in the headlines and people say that their blood samples are taken, taken to US – I don’t know. I don’t know what is in their mind. But there is always controversy. So to come with a polypill, it may be, in that sense there may be some issues. (Cardiologist)

Within the inner setting, nurses noted the importance of informal tele-communication channels such as WhatsApp text messages in promoting medication refills, medication adherence through text-message reminders, and retention in clinical research.

#### Individuals and implementation process

The HFrEF polypill innovation recipients, patients, alluded to the importance of adjunctive and complementary therapies including yoga to their overall health.

Lifestyle means right from the beginning I have a strict and disciplined lifestyle. I do some Yoga and then I do simple exercises and main thing is music is my passion. So whatever it is, be it any tension or any problem I take care of it with music. I play the harmonium, then I sing songs, so whatever problem is there I get rid of it with music. (Patient)

Opinion leaders and HFrEF polypill innovation deliverers, cardiologists, emphasized the distinct benefits of HFrEF polypills and expressed a strong interest in integrating this innovation into their healthcare systems highlighting the appropriateness of this strategy.

So I personally think this should have come much, much, much earlier, this idea of polypill should have come much, much, much earlier. (Cardiologist)

### Implementation Research Logic Model

**Figure 2:**
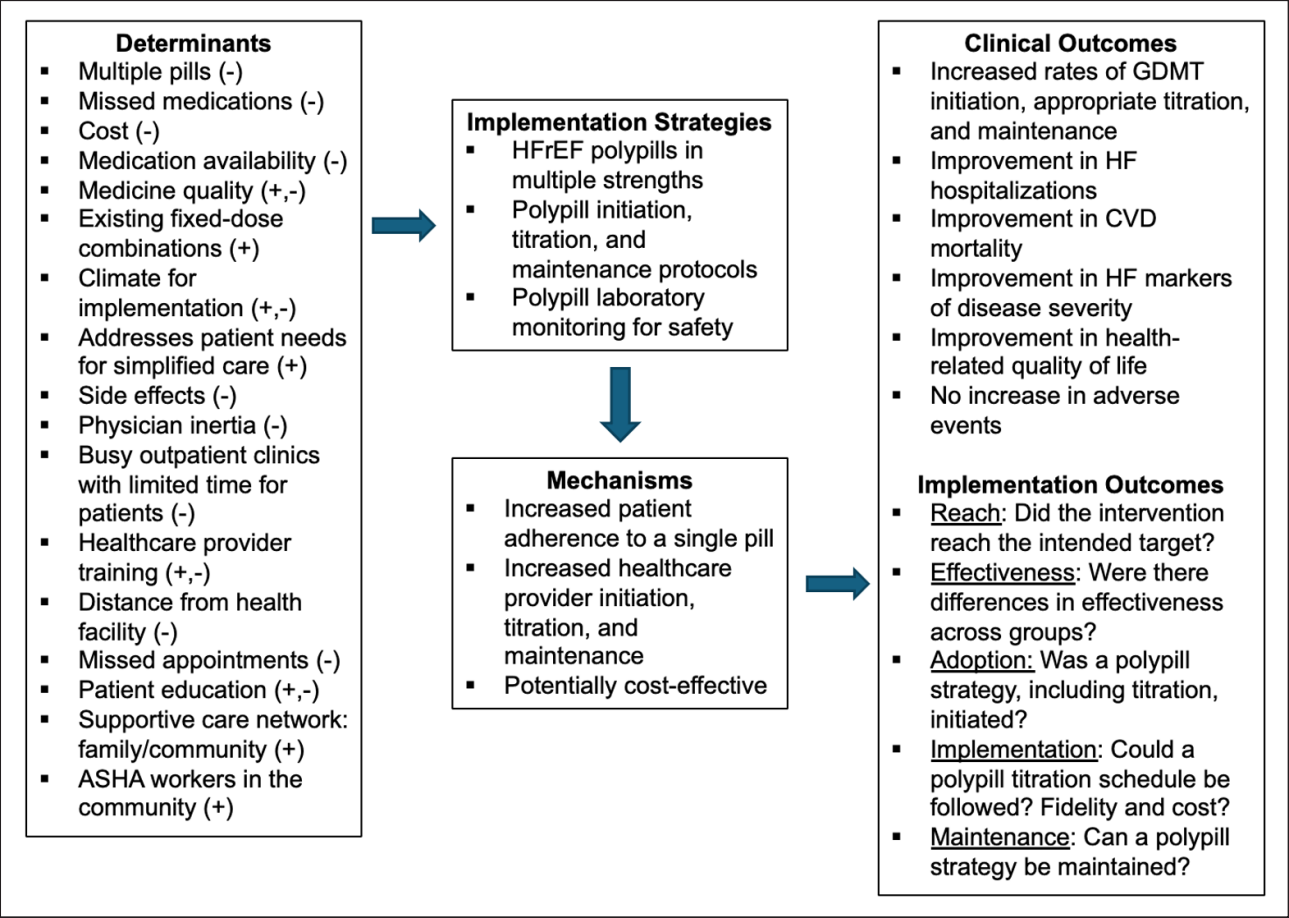
Implementation Research Logic Model for HFrEF polypill-based implementation strategy in India (14). **Abbreviations:** HFrEF: heart failure with reduced ejection fraction; ASHA: Accredited Social Health Activist; GDMT: guideline-directed medical therapy; HF: heart failure; CVD: cardiovascular disease. Positive valence (+): facilitator. Negative valence (-): barrier.

The Implementation Research Logic Model was developed by Smith and colleagues to understand the connections between determinants (barriers or facilitators of implementation), implementation strategies (interventions), mechanisms (processes by which the implementation strategies operate to lead to desired outcomes), and outcomes (clinical, intermediate service outcomes, and implementation) for a study or project (14). We used a mixed methods approach integrating qualitative data from the key informant in-depth interviews with quantitative data from stakeholder surveys to identify determinants of implementation of a HFrEF polypill-based strategy in India including both facilitators (e.g., addresses patient needs for simplified care) and barriers (e.g., patient distance from a health facility, beliefs) outlined in the Implementation Research Logic Model (Figure 2). The proposed implementation strategies include 1) HFrEF polypills, 2) HFrEF polypill initiation, titration, and maintenance protocols, and 3) HFrEF polypill laboratory monitoring protocols for safety which we postulate will lead to desired clinical and implementation outcomes through multiple mechanisms including increased medication adherence to a single pill (Figure 2). The determinants were elucidated from the qualitative and quantitative data collected, whereas the implementation strategies, proposed mechanisms, and implementation outcomes are based on investigator judgment.

## Discussion

This convergent parallel mixed methods study provides insights into the facilitators, barriers, and context of heart failure care in India, and explores physician, nurse, pharmacist, and patient perspectives on a HFrEF polypill implementation strategy in India. Physicians and cardiologists representing most states and union territories in India noted moderate acceptability, appropriateness, and feasibility of the proposed HFrEF polypill implementation strategy. In-depth interview participants highlighted numerous relative advantages of the HFrEF polypill innovation including potential to simplify medication regimens and improve patient adherence. Key relative disadvantages elucidated include concerns about side effects and interruption of multiple GDMT medications due to polypill discontinuation for side effects or hospitalizations. The Implementation Research Logic Model integrates both quantitative and qualitative results to provide a theoretical framework for future HFrEF polypill research in South Asia.

The National Heart Failure Registry of India is a facility-based clinical registry of 10,851 consecutive patients hospitalized with acute decompensated heart failure in 53 hospitals across 21 different states. Of the eligible HFrEF patients in the registry, only 47.5% received GDMT (15). Importantly, those who did not receive GDMT experienced higher mortality compared to patients who received GDMT, revealing a key target for intervention with a HFrEF polypill-based implementation strategy. Previous qualitative research on heart failure care in Kerala, India identified patient non-adherence to GDMT as well as challenges with initiation and titration of GDMT by physicians due to busy clinical practices as important barriers to optimal heart failure care which aligns with our research findings (16,17). An interrupted time series study evaluating a hospital-based quality improvement intervention including discharge checklists, audit-and-feedback mechanisms, and patient education demonstrated that patients had 70% higher odds of receiving GDMT at discharge in the intervention period compared to the control period (41% vs 28%, adjusted odds ratio [aOR] = 1.70, 95% CI 1.17, 2.46) (18). Despite this improvement, more than half of eligible patients with HFrEF remained sub-optimally treated suggesting additional implementation strategies are needed to improve care for this population. Future data from an ongoing trial evaluating collaborative care models delivering integrated heart failure care led by a trained nurse with support from physicians, dieticians, physiotherapists, and clinical psychologists to improve clinical outcomes of patients with HFrEF will inform outpatient and longitudinal heart failure care in India (19). A HFrEF polypill implementation strategy could be an important complement to existing and future strategies to improve GDMT use and HF care in South Asia.

Cost was, highlighted as a key characteristic of HFrEF polypills, identified by physicians as being important to their patients in considering acceptability, appropriateness, and feasibility of a HFrEF polypill-based implementation strategy in India. There is wide variability in availability, price, and affordability of GDMT in both public and private sectors of low- and middle-income countries (20). However, the robust generic drug market in India has led to greater accessibility and affordability of essential medicines including GDMT for patients with heart failure. A HFrEF polypill-based implementation strategy will need to be cost-effective to be implemented, adopted, scaled, and sustained. This study highlights the use of existing, but sub-optimal, fixed-dose combinations such as ACE-I and beta-blocker by physicians in India demonstrating acceptability of combination polypill therapy for the treatment of HFrEF patients, which has important policy implications and aligns with previous research on polypills for cardiovascular disease in India. Important considerations for future trials evaluating a HFrEF polypill implementation strategy include accompanying laboratory monitoring protocols to emphasize safety and initiation, titration, and maintenance protocols to guide physicians. Type I or Type II hybrid-effectiveness trial designs which emphasize *a priori* efficacy, effectiveness, and implementation process are ideally suited to study a HFrEF polypill-based implementation strategy in India and other undertreated populations globally (21).

This study has several strengths including the convergent parallel mixed methods data collection integrating both quantitative data from stakeholder surveys across India to members of the Cardiological Society of India (the largest professional association of cardiologists in the country) and qualitative data from key informant in-depth interviews to understand diverse perspectives on a HFrEF polypill-based implementation strategy to inform future clinical trial design (8). Furthermore, this is one of the few studies harnessing qualitative research methods to understand perspectives from patients with heart failure in South Asia, strengthening the applicability of our findings. Key limitations include limited sample size for the electronic stakeholder survey, greatest representation of private practice cardiologists in the survey, and selection of majority of participants for the key informant in-depth interviews from the state of Kerala in South India which could introduce selection bias. In addition, in-depth interview transcripts were not returned to participants for comments and/or corrections. Despite these limitations, our study is the first, to our knowledge, to understand diverse perspectives on a HFrEF polypill-based implementation strategy to directly assist future research in South Asia.

## Conclusion

This convergent parallel mixed methods study demonstrates that a HFrEF polypill-based implementation strategy is considered moderately acceptable, feasible, and appropriate among healthcare providers in India. We identified facilitators and barriers to design contextually relevant implementation strategies including HFrEF polypills outlined in an Implementation Research Logic Model to inform future research to improve heart failure trials and care in South Asia and beyond.

## Additional Files

The additional files for this article can be found as follows:

10.5334/gh.1348.s1 Appendix 1. HFrEF polypill stakeholder survey with modified implementation science outcome measures.

10.5334/gh.1348.s2 Appendix 2. Semi-structured interview guides for stakeholders, health professionals, and patients.

10.5334/gh.1348.s3 Appendix 3. Consolidated Criteria for Reporting Qualitative Research (COREQ).
