# Supplementary material for: Heart Failure With Reduced Ejection Fraction Polypill Implementation Strategy in India: A Convergent Parallel Mixed Methods Study
Source: Glob Heart. 2024 Aug 26;19(1):69. doi: 10.5334/gh.1348 (PMC11363896; doi:10.5334/gh.1348)
Supplement: Appendix 1. — HFrEF polypill stakeholder survey with modified implementation science outcome measures. [file gh-19-1-1348-s1.pdf]

Appendix 1: HFrEF polypill stakeholder survey with modified implementation science outcome measures

Heart Failure Polypill Survey

Key Information About This Research Study

The purpose of this study is to better understand heart failure care in India and the potential use of a polypill for patients with heart failure with reduced ejection fraction (HFrEF). You will be asked to complete an online survey that will take approximately 10 minutes to complete and will include questions regarding your thoughts on a HFrEF polypill. There is minimal risk to you of participation in this study. Your responses will be anonymized and your name will not be associated with your responses. The main benefit is that your responses will help the study team better understand how to implement a HFrEF polypill in India. You will not be compensated for participation in this study.
This research is supported by Northwestern University.

ELECTRONIC CONSENT: Please consent by clicking the button below in order to proceed to the survey. Thank you!

□ I CONSENT TO PARTICIPATE IN THIS SURVEY

HFrEF Polypill Survey

Please fill out the survey below on a heart failure with reduced ejection fraction (HFrEF) polypill. The information you provide will be used for the purpose of this research study. Your confidentiality will be maintained as no names will be associated to your responses. The survey will be destroyed after completion of the study.

Our goal is to create a polypill for patients that have heart failure with reduced ejection fraction (HFrEF).

A HFrEF polypill would be one pill containing the necessary medications for HFrEF instead of the many different pills most patients currently take for heart failure.

A HFrEF polypill would be one pill that contains:

ACE-I or ARB or ARNI Beta-blocker Mineralocorticoid receptor antagonist Sodium-glucose co-transporter 2 inhibitor (SGLT2i) We would like to know your opinion about the use of a HFrEF polypill once it is developed in your clinical practice.

1. How much of a problem is taking multiple pills daily for your patients with HFrEF?	☐Large problem<br>☐ Moderate problem<br>☐ Minor problem<br>☐ Not a problem
2. What are the most important characteristics of a HFrEF polypill that your patients will care about? Please check all that apply.	☐ Size of the HFrEF polypill<br>☐ Cost of the HFrEF polypill<br>☐ Side effects of the HFrEF polypill<br>☐ Once daily dosing of the HFrEF polypill<br>☐ Efficacy of the HFrEF polypill<br>(Choose all that apply)

*The following questions have 5 options of responses to choose from (Completely disagree, Disagree, Neither agree nor disagree, Agree, Completely agree)

3. A HFrEF polypill meets my approval.
4. A HFrEF polypill seems fitting for my patients.
5. I think I would be able to use a HFrEF polypill in my clinical practice.
6. A HFrEF polypill would be appealing to my patients.
7. A HFrEF polypill seems suitable for my patients

8. Using HFrEF polypills for my patients seems possible once developed.
9. I like the idea of a HFrEF polypill.
10. A HFrEF polypill seems applicable to my patients.
11. Using a HFrEF polypill seems doable for patients.
12. I welcome a HFrEF polypill as an additional treatment option for my patients with HFrEF.
13. A HFrEF polypill seems like a good match for my patients.
14. A HFrEF polypill seems easy to use for me and my patients.

DEMOGRAPHICS

15. What is your age? (in years)
16. What is your sex? (Male, Female, or Other)
17. What is your job?
18. How many years have you been working in healthcare?
19. What type of healthcare setting do you work in? (Public, Private, Other)
20. Which state/union territory do you live in?

---

21. Please share any comments and thoughts you have on the idea of a HFrEF polypill.
