# Supplementary material for: Heart Failure With Reduced Ejection Fraction Polypill Implementation Strategy in India: A Convergent Parallel Mixed Methods Study
Source: Glob Heart. 2024 Aug 26;19(1):69. doi: 10.5334/gh.1348 (PMC11363896; doi:10.5334/gh.1348)
Supplement: Appendix 2. — Semi-structured interview guides for stakeholders, health professionals, and patients. [file gh-19-1-1348-s2.pdf]

Appendix 2: Semi-structured interview guides for stakeholders, health professionals, and patients

Stakeholder In-Depth Interview Guide

<u>Introduction script:</u>

Thank you for your time and agreeing to participate in this interview. This project is being conducted by researchers from Cardiological Society of India-Kerala and Northwestern University. The objective of this interview is to gain more insights into the perceptions and potential use of a polypill for patients with heart failure with reduced ejection fraction.

This interview will be recorded and the information you provide will be used for the purpose of this research study. Your confidentiality will be maintained as no names will be associated to your responses. The recorded audio and video files will be destroyed after completion of the study.

[Turn on recording on phone or video-conference call]

Part 1: Outer and inner setting

1. How do you learn about the needs of patients served by your hospital or healthcare organization?

2. What strategies, policies or incentives do you have to implement new research studies?

3. Which individuals in your hospital or healthcare organization are essential in making decisions about being involved in new research studies?

(Probes: Explore who needs to support this intervention to be successfully implemented)

4. How does your hospital or healthcare organization communicate to doctors about new research studies that they can refer patients to?

5. How would you describe the culture of your hospital or healthcare organization to new research studies and ideas?

(Probes: Explore acceptability of new studies)

6. What are the important factors at your hospital or healthcare organization in implementing a new research study successfully?

7. How do you learn about new research findings?

8. If these research findings are beneficial to the patients your organization serves, how do you disseminate that information?

9. What are your thoughts on clinical trials?

(Probes: Explore attitudes towards pharmacological clinical trials)

Part 2: Process

<u>Script:</u>

We aim to create a polypill for patients with heart failure with reduced ejection fraction that would be <u>one pill</u> containing three different medications (i.e. an ACE-I or ARB, beta-blocker and aldosterone antagonist).

10. How would you like to be involved in the planning and implementation of a study to evaluate whether a heart failure polypill may be beneficial for patients?

11. Who are the expert opinion leaders on heart failure in your hospital, healthcare organization, and/or state?

<u>Script:</u>

In the proposed study to evaluate the heart failure polypill, we aim to provide patients receiving the intervention of the heart failure polypill <u>free of charge for the duration of the study</u>. We also aim to provide patients receiving usual care their usual medications for heart failure <u>free of charge for the duration of the study</u>.

12. What are your thoughts on this?

(Probes: Explore major challenges or barriers, facilitators or strategies that will help, variation across public or private hospitals)

Script:

After the study is completed, the patients who are receiving the heart failure polypill will transition back to usual medications for heart failure.

13. Tell me your thoughts on how possible it will be to implement this.

(Probes: Explore major challenges or barriers, facilitators or strategies that will help, variation across public or private hospitals)

Conclusion script:

Thank you so much for your time and providing information for this research study.

[Turn off recording on phone or video-conference call]

Health Professional In-Depth Interview Guide

Introduction script:

Thank you for your time and agreeing to participate in this interview. This project is being conducted by researchers from Cardiological Society of India-Kerala and Northwestern University. The objective of this interview is to gain more insights into the perceptions and potential use of a polypill for patients with heart failure with reduced ejection fraction.

This interview will be recorded and the information you provide will be used for the purpose of this research study. Your confidentiality will be maintained as no names will be associated to your responses. The recorded audio and video files will be destroyed after completion of the study.

[Turn on recording on phone or video-conference call]

Part 1: Intervention

1. What is your usual approach to treating patients with heart failure with reduced ejection fraction?

(Probes: Which drug do you start first and why? Do you prefer escalating doses of one drug class before adding a second drug class of guideline-directed medical therapy?)

2. What are some facilitators to getting patients with heart failure with reduced ejection fraction on guideline-directed medical therapy?

3. What are some barriers to getting patients with heart failure with reduced ejection fraction on guideline-directed medical therapy?

(Probes: Consider prescribing practices amongst physicians, challenges obtaining medications from pharmacies due to stockouts or falsified medications or cost, patient lost-to follow-up after initial hospitalization or clinic visit)

Script:

A polypill is one pill that will include multiple medications for patients with heart failure with reduced ejection fraction including an ACE-I or ARB, beta-blocker, and aldosterone antagonist.

4. Which types of patients that you take care of do you think would benefit from a heart failure polypill? Which types of patients do you think may not benefit?

(Probes: Consider compliance, severity of heart failure, distance from health facility)

5. Thinking about your patients, would you consider starting a polypill in patients newly diagnosed with heart failure with reduced ejection fraction? Why or why not?

6. Would you consider switching your patients to a heart failure polypill at any stage of treatment? Why or why not?

7. What evidence would you want before prescribing a heart failure polypill?

8. What may be the relative advantage of a heart failure polypill over usual care practices for patients with heart failure with reduced ejection fraction? Why?

(Probes: Adaptability, trialability, complexity, cost)

9. What are your thoughts on a heart failure polypill? *(Probes: Consider knowledge, beliefs, and attitudes regarding acceptability of the intervention)* What would be the most important quality of the polypill?

Part 2: Outer and inner setting

<u>Probes:</u>

10. Have you been part of a clinical trial in the past as a researcher? How was your experience?

(Probes: Any challenges with participant recruitment and retention, attitudes at clinic/hospital level to doing clinical research, opinion leaders/champion)

11. What strategies, policies or incentives would motivate you to prescribe a heart failure polypill to your patients instead of current usual care?

(Probes: cost)

12. Who would be essential in making decisions about how the study is designed?

(Probes: Explore who needs to support this intervention to be successfully implemented)

13. What are the important factors at your hospital or healthcare organization in implementing a new intervention (such as a heart failure polypill) successfully?

(Probes: opinion leader? policy? patients attitudes?)

14. If a heart failure polypill was available, what kind of education would providers need?

Part 3: Process

15. How would you like to be involved in the planning and implementation of a study to evaluate whether a heart failure polypill may be beneficial for your patients? Would you be interested in enrolling someone in this study and what would make this study more attractive?

16. Who are the expert opinion leaders on heart failure in your hospital, healthcare organization, and/or state?

<u>Script:</u>

In the proposed study, we want to compare the heart failure polypill to usual care. We will provide patients receiving the intervention of the heart failure polypill <u>free of charge for the duration of the study</u>. We propose to provide patients receiving usual care their usual medications for heart failure <u>free of charge for the duration of the study</u>.

22. What are your thoughts on this?

(Probes: Explore major challenges or barriers, facilitators or strategies that will help, variation across public or private hospitals)

<u>Script:</u>

After the study is completed, the patients who are receiving the heart failure polypill will transition back to usual medications for heart failure.

23. What may be the challenges to this approach?

(Explore major challenges or barriers, facilitators or strategies that will help, variation across public or private hospitals)

Part 4: Covid-19

24. How has the Covid-19 pandemic affected your clinical practice and management of heart failure patients?

(less people coming in, etc.)

Conclusion script:

Thank you so much for your time and providing information for this research study.

[Turn off recording on phone or video-conference call]

Patient In-Depth Interview Guide

Introduction script:

Thank you for your time and agreeing to participate in this interview. This project is being conducted by researchers from Cardiological Society of India-Kerala and Northwestern University. The objective of this interview is to gain more insights into the perceptions and potential use of a polypill for patients with heart failure with reduced ejection fraction.

This interview will be recorded and the information you provide will be used for the purpose of this research study. Your confidentiality will be maintained as no names will be associated to your responses. The recorded audio and video files will be destroyed after completion of the study.

[Turn on recording on phone or video-conference call]

Part 1: Current treatment and individual

1. How long have you had heart failure?

2. How has the diagnosis of heart failure affected you?

3. How frequently do you see a doctor for your heart failure?

(Probes: Explore whether they miss any appointments or have any trouble getting to see the doctor)

4. What medications are you taking for heart failure? Describe any challenges you may have in obtaining these medications (i.e. travel to pharmacy, cost) or taking these medications every day (i.e. adherence, number of pills, side effects).

5. How do you feel after taking these medications? Do you think these medications have helped? If so, how have they helped?

Part 2: Intervention and process

Script:

We aim to create a polypill for patients that have heart failure like you. A polypill would be one pill containing all the necessary medications for heart failure instead of the three different pills many patients currently take for heart failure.

6. Tell us your thoughts on taking one pill daily for your heart failure instead of the many different pills you may be taking for your heart failure.

(Probes: Explore potential benefits and concerns)

Script:

We would like to study whether one pill containing all the necessary medications for heart failure make people feel better compared to the three different pills many patients with heart failure currently take. In this study, half of the participants in the study would receive one pill and the other half of the participants would continue their usual medications for heart failure. Participants who receive one pill will be selected randomly.

7. Would you be interested in participating in such a study? Why or why not?

(Probes: Explore thoughts and potential concerns)

We would like to provide all participants their medications for heart failure free of charge during the study which will last 1 year. After the study is completed, all participants will need to resume obtaining and paying for their medications for heart failure as they did before the study.

8. Would you be interested in participating in such a study? Why or why not?

(Probes: Explore thoughts and potential concerns)

During the study, you may need to travel to come to outpatient department appointments where you usually receive your heart failure care up to 4 times over one year. This may be more frequent than you normally see your heart failure doctor in one year.

9. Is this feasible for you?

(Probes: Explore how far the outpatient department is, how much time/cost it takes to travel, and what would make it easier?)

10. What additional type of information would you need to make the decision to participate in such a study?

11. What would be important besides yourself in your decision to participate or not?

12. Have you ever been in a research study before?

(Probes: If yes, explore benefits and challenges of participation)

13. What motivates you to potentially participate (or not participate) in the study described?

Part 3: Covid-19 Pandemic

14. How has the Covid-19 pandemic affected your heart failure care?

(Probes: Challenges in traveling to see your doctor, cost of medications, anxiety)

<u>Conclusion script:</u>

Thank you so much for your time and providing information for this research study.

[Turn off recording on phone or video-conference call]
